# A Physiological Approach to Assessment and Rehabilitation of Acute Concussion in Collegiate and Professional Athletes

**DOI:** 10.3389/fneur.2018.01115

**Published:** 2018-12-20

**Authors:** Michael J. Ellis, John Leddy, Dean Cordingley, Barry Willer

**Affiliations:** ^1^Section of Neurosurgery, Department of Surgery, Pediatrics and Child Health, Children's Hospital Research Institute of Manitoba, Canada North Concussion Network, University of Manitoba, Winnipeg, MB, Canada; ^2^Pan Am Concussion Program, Winnipeg, MB, Canada; ^3^UBMD Department of Orthopaedics and Sports Medicine, Buffalo, NY, United States; ^4^Pan Am Clinic Foundation, Winnipeg, MB, Canada; ^5^Department of Psychiatry, Jacobs School of Medicine and Biomedical Sciences, State University of New York at Buffalo, Buffalo, NY, United States

**Keywords:** acute concussion, physiology, assessment, targeted rehabilitation, collegiate and professional athletes

## Abstract

Sport-related concussion is an important condition that can affect collegiate and professional athletes. Expert consensus guidelines currently suggest that all athletes who sustain acute concussion be managed with a conservative approach consisting of relative rest and gradual resumption of school and sport activities with active intervention reserved for those with persistent post-concussion symptoms lasting >10–14 days for adults. Unfortunately, these recommendations place little emphasis on the rapid physical deconditioning that occurs in athletes within days of exercise cessation or the pathophysiological processes responsible for acute concussion symptoms that can be successfully targeted by evidence-based rehabilitation strategies. Based on our evolving approach to patients with persistent post-concussion symptoms, we now present an updated physiological approach to the initial medical assessment, rehabilitation, and multi-disciplinary management of collegiate and professional athletes with acute concussion. Utilizing the results of a careful clinical history, comprehensive physical examination and graded aerobic exercise testing, we outline how team physicians, and athletic training staff can partner with multi-disciplinary experts in traumatic brain injury to develop individually tailored rehabilitation programs that target the main physiological causes of acute concussion symptoms (autonomic nervous system dysfunction/exercise intolerance, vestibulo-ocular dysfunction, and cervical spine dysfunction) while maintaining the athlete's physical fitness during the recovery period. Considerations for multi-disciplinary medical clearance of collegiate and professional athletes as well as the application of this approach to non-elite athletes are also discussed.

Concussion is a form of traumatic brain injury (TBI) that has become an increasingly recognized condition among collegiate and professional athletes participating in contact and collision sports. Clinical research indicates that the majority of adult athletes achieve a complete neurological and neurocognitive recovery within 10–14 days of injury, with ~10–15% experiencing symptoms that persist beyond this timeframe (1, 2). Historically, expert consensus guidelines have recommended that athletes with acute sport-related concussion restrict their physical and cognitive activity until they are symptom-free and then initiate a gradual return to physical exercise and sport-specific activities (2–5). Although more recent guidelines advocate for an earlier return to sub-symptom threshold physical and cognitive activity (6), they offer limited guidance for the management of collegiate and professional athletes who undergo rapid physiological deconditioning within days of exercise cessation and are expected to return to optimal sport performance immediately following medical clearance. Furthermore, these guidelines do not take into consideration the heterogeneous pathophysiological processes that account for the clinical manifestations of acute concussion that can be successfully targeted with established and emerging evidence-based rehabilitation strategies.

Based on the principles of our rapidly evolving physiological approach to management of patients with persistent post-concussion symptoms (PPCS) (7–13), we now introduce a novel and up-to-date approach to the initial assessment, rehabilitation, and multi-disciplinary management of collegiate and professional athletes with acute concussion. Drawing on evidence from the general sport-related concussion literature and our own personal experience, we present the hypothesis that the clinical manifestations of acute concussion are caused by heterogeneous pathophysiological processes that can be identified by a comprehensive medical assessment including a careful clinical history, complete physical examination, and graded aerobic exercise testing. Team physicians and athletic training staff can use the results of this assessment to develop an individually tailored rehabilitation program that promotes the active recovery and treatment of acute concussion while minimizing physiological deconditioning.

## The Physiology and Clinical Manifestations of Acute Concussion

Basic science research suggests that acute concussion is characterized by a metabolic energy crisis that arises as a consequence of a mismatch between cellular energy demands and impairments in energy substrate delivery. Animal model studies suggest that biomechanical forces applied to the brain result in a cascade of cellular events including acute alterations in neuronal depolarization, excitatory neurotransmitter release, cellular ion homeostasis, glycolysis, and oxidative functioning as well as disturbances in cerebral blood flow regulation (14–16). In humans, advanced neuroimaging, and laboratory studies have demonstrated that acute concussion is associated with alterations in cerebral metabolism, resting cerebral blood flow, cerebrovascular reactivity, and neurovascular coupling that in some cases may persist beyond symptomatic recovery (17–24). Although the mechanisms underlying these cerebrovascular disturbances remain to be fully elucidated, studies suggest that impairments in autonomic nervous system functioning may play an important role. Indeed, an accumulating body of literature suggests that acute concussion is associated with impairments in autonomic nervous system functioning (25–36) that may mediate concussion symptoms and exercise intolerance via aberrant neuromodulatory control of cerebral blood flow (37, 38).

Patients with acute concussion present with a wide spectrum of neurological symptoms that reflect these global alterations in brain physiology. Symptoms commonly reported during the acute phase of injury include headache, nausea, dizziness, light and sound sensitivity, fatigue, drowsiness, sleep disturbance, and difficulty with memory and concentration. As a consequence of the metabolic energy crisis that characterizes this injury, patients with acute concussion often experience worsening of their symptoms during periods of increased physical and cognitive exertion. Patients with acute concussion can present with an elevated resting heart rate and evidence of orthostatic hypotension while graded aerobic exercise testing often reveals evidence of early exercise intolerance (39).

In addition to the generalized symptoms and exercise intolerance observed among patients with acute concussion, abnormal biomechanical forces transmitted to the head and neck can also lead to physiological disturbances in vestibulo-ocular and cervical spine functioning that can result in a unique constellation of symptoms and objective physical examination findings. Impairments in vision, oculomotor, and vestibular functioning can occur as a result of structural or functional injury to the brain or peripheral visual or vestibular sensory organs (40–42). Patients with vestibulo-ocular dysfunction typically present with intermittent blurred vision or diplopia, dizziness, difficulty focusing, vertigo, motion sensitivity, postural imbalance, and headaches that are provoked by prolonged periods of visual or vestibular stimulation. Physical examination commonly reveals a combination of findings including objective impairments in convergence, accommodation, smooth pursuits, saccades, balance, gait, and vestibulo-ocular reflex functioning (43, 44). In more rare cases, athletes with sport-related concussion can present with cranial neuropathies (45, 46) or clinical evidence of peripheral vestibular disorders, such as benign paroxysmal positional vertigo (BPPV) or unilateral vestibular hypofunction (47–49). Studies suggest that acute concussion patients who present with clinical evidence of vestibulo-ocular dysfunction at initial assessment report a higher burden of concussion symptoms and take longer to recover compared to those without these features (50, 51). Balance, gaze stabilization, and head and body coordination are also dependent on highly integrated sensorimotor pathways involving the cervical spine. Biomechanical injury to the cervical spine including central and peripheral components of the cervicocollic, vestibulocollic, and cervicoocular reflexes can lead to functional disturbances that manifest as dizziness, vertigo, visual disturbance, gait instability, motion sensitivity, fogginess, and postural imbalance (42, 52). These forces can also lead to local tissue inflammation and sensitization of nocioceptive pathways giving rise to neck pain, and cervicogenic headaches that radiate to the posterior aspect of the scalp, temples, and orbits and are often exacerbated by activities requiring prolonged neck stabilization (53–55). Common physical examination findings in acute concussion patients with cervical spine dysfunction include reduced cervical spine range of motion; tenderness and spasm of the sternocleidomastoid, scalene, paraspinal, and sub-occipital muscles; hyper- or hypo-mobility of the alar ligaments and dizziness or vertigo elicited during provocative cervical spine testing (56–58). Similar to those with vestibulo-ocular dysfunction, acute concussion patients with evidence of cervical spine dysfunction present with a greater burden of concussion symptoms and take longer to recover compared to those without these clinical findings (59).

Taken together, there is emerging evidence that acute concussion can result in a wide spectrum of clinical symptoms and examination findings that are reflective of heterogeneous and often overlapping physiological processes that have an important impact on patient recovery. While conservative management with physical and cognitive rest will lead to symptom resolution in the vast majority of acute concussion patients, this approach is associated with its own negative effects on physiological functioning, especially when applied to collegiate and professional athletes.

## The Physiology and Clinical Manifestations of Physical Deconditioning

Regular exposure to physical exercise through training, practice, and game play leads to a number of physiological adaptations that help optimize physical fitness and performance among athletes. However, abrupt cessation of physical exercise, such as during injury, illness, or due to other factors, can lead to rapid declines in cardiorespiratory and metabolic functioning and pronounced performance loss (60). Experimental studies suggest that even brief periods of restricted physical inactivity or bed rest can have negative effects on cardiovascular, musculoskeletal, metabolic, endocrine, hematologic, and psychological functioning (61) and can result in symptoms (light headedness, fatigue, dizziness, irritability etc.) that are similar to those observed among patients with acute concussion. Among the systems that undergo changes within weeks of physical exercise cessation in athletes is the autonomic nervous system. Research suggests that exercise cessation is associated with reduced total blood volume and cardiac stroke volume. The autonomic nervous system compensates for these changes in stroke volume by increasing sympathetic activity, and in turn heart rate, to maintain oxygen delivery during sub-maximal exercise (62). After as little as 3 days of bed rest regularly exercising endurance athletes have been found to exhibit reductions in peak exercise performance and impaired neuroendocrine responses to graded exercise (63). To help mitigate these changes, studies suggest that individually tailored exercise programs can be used to provide a stimulus to maintain exercise-induced physiological adaptions and fitness levels during periods of relative physical inactivity (64).

## Expert Consensus Approaches to Acute Concussion Management

Despite ongoing scientific advances in concussion and sport physiology over the past three decades, published expert guidelines continue to advocate for a uniform and largely conservative approach to management of acute concussion in athletes. Historically, experts have held the view that acute concussion is a condition characterized by non-specific neurological symptoms that follow a sequential course to spontaneous resolution and are reflective of temporary global alterations in brain functioning. Consistent with this understanding, consensus guidelines published up to 2013 by numerous groups have uniformly recommended that all athletes with acute sport-related concussion be managed with strict physical and cognitive rest until all symptoms completely resolved followed by structured re-exposure to physical exercise and sport-specific activities (2–5). Following this time period, newer research emerged to suggest that prolonged periods of prescribed physical and cognitive rest may have a detrimental effect on patient outcomes (65–67) and that patients with acute concussion who participated in higher (but moderate) levels of physical activity experienced a lower risk of developing PPCS compared to those who participated in lower levels of activity (68). Consequently, the 5th International Consensus Statement on Concussion in Sport published in 2017 advocated for a modified approach whereby acute concussion patients should be managed with a brief period (24–48 h) of rest followed by initiation of symptom-limited activity with the goal of gradual reintroduction of school and sport activities (6). Although these authors acknowledge that concussion patients may benefit from a multi-modal assessment and individualized treatment plan targeting physical and psychological factors, these considerations appear to apply only to those who experience PPCS, which is defined as symptoms that persist beyond 10–14 days in adults and >4 weeks in youth. Because these authors recommend that all elite and non-elite athletes be managed using the same management principles and provide no recommendations to quantify exercise intolerance in acute concussion patients (6), this approach carries the risk of returning collegiate and professional athletes who have recovered from an acute concussion to highly demanding sport settings in a state of relative physiological deconditioning.

In addition to these published guidelines, other authors have proposed alternative approaches to concussion management. For instance, authors from the University of Pittsburgh have presented a comprehensive targeted approach to the clinical care of sport-related concussion in athletes. Relying largely on symptom inventories and abbreviated vestibular/oculomotor and computerized neurocognitive screening tools, the authors suggest that athletes with sport-related concussion can be classified into symptom-based trajectories or profiles (69, 70). Unfortunately, this approach places little emphasis on the medical assessment of athletes presenting with acute head and neck trauma, the heterogeneous pathophysiological causes of concussion symptoms, or takes into consideration the physiological impact of acute deconditioning in individual athletes. Consequently, we believe there is an unmet need for a more comprehensive, physiologically-relevant approach to the initial medical assessment and multi-disciplinary management of collegiate and professional athletes with acute concussion.

### A Physiological Approach to Acute Concussion Assessment and Management

**Figure 1:**
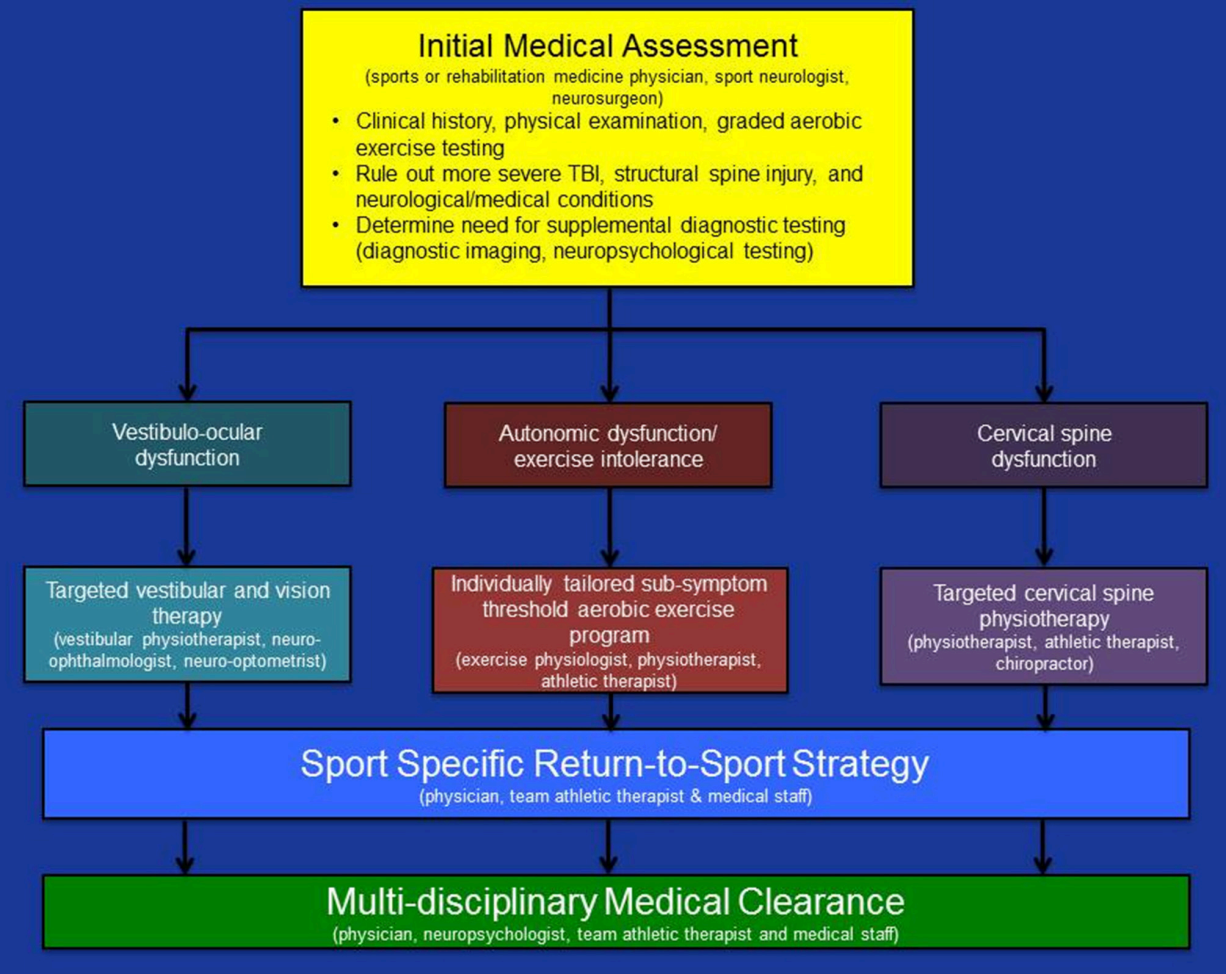
Summary of a physiological approach to medical assessment and multi-disciplinary management of acute concussion in collegiate and professional athletes. Updated from Ellis et al. (10).

The initial medical assessment of all collegiate and professional athletes with suspected acute concussion must achieve three important objectives: (1) provide a definitive medical diagnosis of acute concussion; (2) identify the clinico-pathophysiological features that may place the athlete at risk of prolonged recovery; and (3) inform the development of an individually tailored rehabilitation program that targets the pathophysiological causes of concussion symptoms while maintaining the athlete's physical fitness level during the recovery process (see Figure 1).

In order to diagnose an acute concussion an experienced physician must reliably rule out more severe forms of TBI, structural injuries to the cervical spine, as well as medical, and neurological conditions that can present with concussion-like symptoms (6, 71, 72). All collegiate and professional athletes with suspected acute concussion should undergo initial medical assessment by an experienced sports or rehabilitation medicine physician, sport neurologist, or neurosurgeon who is trained and licensed to independently complete a thorough clinical history, perform a comprehensive physical examination, and order and interpret supplemental diagnostic tests as needed. In many cases, physicians will need to partner with multi-disciplinary experts to confirm the diagnosis of acute concussion, facilitate individually tailored rehabilitation programs, and confirm physiological and neurocognitive recovery. These individualized rehabilitation programs must also be structured and paced in such a way as to account for team practice and travel schedules as well as the implementation of mandatory aspects of standardized league-wide concussion protocols, such as post-injury formal neuropsychological testing (73–75).

### Clinical History

Injury details that are important to collect during the clinical history include the setting and mechanism of injury, initial concussion symptoms, and whether the patient experienced a loss of consciousness, post-traumatic antero- or retrograde amnesia, neck pain, focal neurological deficits, such as weakness or numbness of the extremities or loss of vision, as well as convulsions at the time of injury. Documentation from the team medical and athletic therapy staff regarding immediate sideline assessments, previous medical care (including diagnostic imaging studies) as well as any video footage of the injury should also be obtained and reviewed. Clinical research and systematic reviews suggest that a number of pre-injury and non-modifiable risk factors may place athletes at an elevated risk of prolonged recovery following concussion (76). Information should be sought regarding a history of previous concussions including the date, initial injury characteristics, and symptoms, length of recovery, previous treatments, and consultations as well as the results of previous diagnostic tests or imaging studies associated with these injuries. In addition, patients should be asked about past history and treatment for conditions, such as attention-deficit hyperactivity disorder, migraine, or non-specific headaches, learning disorders, mood disorders, such as depression or anxiety, as well as neuro-ophthalmological conditions (i.e., strabismus, accommodative disorders). Athletes should be asked about a history of cervical spine injuries or chronic neck stiffness and whether they regularly receive ongoing manual therapy or chiropractic care for baseline cervicogenic symptoms. It is also important to inquire about any other recently diagnosed medical conditions that can potentially contribute to concussion-like symptoms (i.e., infections, hematological disorders, sleep, or mood disorders) as well as collect details regarding a family history of mood disorders, migraine headaches, and neuro-ophthalmological disorders. Current medications and allergies should also be documented as well as smoking history and use of alcohol, recreational drugs, performance-enhancing drugs, and nutritional supplements.

As part of the clinical history, assessment of the patient's symptoms should be undertaken both with the use of a validated symptom inventory, such as the Post-Concussion Symptom Scale that is included as part of the Sport Concussion Assessment Tool-5 (SCAT5) (6), as well as through a more thorough clinical interview. In addition to assessing the magnitude of individual concussion symptoms it is important to inquire about activities or settings that elicit or exacerbate these symptoms (i.e., physical exertion). Finally, it is essential to screen for transient or persistent cervical or focal neurological symptoms (monocular visual disturbance, neck pain, weakness, or numbness of the face or extremities) that may point to co-existing injuries or conditions, such as cranial neuropathies, spine injuries, migraine, or vascular injuries.

### Physical Examination

Following the clinical history all patients with suspected head and spine trauma should undergo a comprehensive physical examination. Although abbreviated tools, such as the SCAT5 (6), King-Devick Test (77) and the Vestibulo-Oculomotor Screening Tool (VOMS) (78) have been recommended as screening tools for athletes with suspected sport-related concussion, use of these tools is insufficient to allow physicians to meet the objectives of an initial medical assessment in athletes with head and neck trauma. The physical examination should include comprehensive assessment of mental status, cranial nerve, motor, sensory, reflex, and cerebellar functioning as well as balance testing (58, 79). Evaluation of vision, oculomotor, and vestibular functioning should include fundoscopic examination, testing of visual fields, and acuity as well as objective assessment of convergence, accommodation, smooth pursuits, and saccades (41). In addition, all patients should undergo a focused cervical spine examination that includes assessment of range of motion, palpation, and when indicated and depending upon level of expertise, supplemental physical examination tests that assess ligamentous integrity and screen for cervicogenic dizziness (i.e., cervical spine flexion test) (56, 57). Patients who report jaw discomfort, temporal headaches, or ear pain should undergo assessment of the jaw and temporo-mandibular joints that includes inspection for jaw deviation and malocclusion, assessment of active jaw movements, palpation, as well as otoscopic examination. Once the presence of an associated cervical spine injury has been clinically excluded, patients may undergo objective testing of the vestibulo-ocular reflex (head thrust testing) as well as a screen for BPPV using the Dix-Hallpike and supine roll tests as clinically indicated (42, 49). In preparation for graded aerobic exercise testing, all patients should undergo measurement of resting blood pressure and heart rate as well as medical screening for any other health conditions that contraindicate exercise testing (8).

### Supplemental Diagnostic Testing

Although the medical diagnosis of acute sport-related concussion can often be made based on the findings of a comprehensive clinical history and physical examination alone, there are instances where the use of supplemental tests are indicated. Given that neuroimaging studies are normal in the vast majority of sport-related concussion patients (80, 81), physicians providing in-office initial medical assessment and follow-up care should only consider these tests in instances where a structural brain or cranial injury is suspected. Magnetic resonance imaging (MRI) of the brain including gradient recalled echo (GRE) and susceptibility-weighted (SWI) sequences should be considered in patients who present with abnormal CT findings, focal neurological deficits, worrisome, or worsening symptoms or post-traumatic seizures (45). Likewise, plain radiographs, or CT imaging should be considered in those with a clinical suspicion of basal or calvarial skull fractures or structural injury to face, orbits, or mandible. Plain radiographs of the cervical spine should be considered in patients with a dangerous mechanism of injury, extremity weakness or parasthesias, delayed onset neck pain, central spine tenderness, and decreased range of motion (82) while CT imaging should be reserved for those with abnormal or inconclusive radiographs. MRI of the cervical spine, including T2 short inversion time recovery (STIR) sequences should be considered in patients presenting with transient or persistent focal neurological deficits localized to the cervical spine to rule out spinal cord injury, nerve root compression, or ligamentous injury (83). Flexion-extension radiographs of the cervical spine should also be considered in patients with clinical evidence of central cord neuropraxia and spinal cord injury without radiographic abnormality (SCIWORA) to rule out dynamic spinal instability (84). Patients with clinical evidence of structural spine injury or instability should be referred to a neurosurgeon or orthopedic spine surgeon for further assessment and management.

In very rare instances when the treating physician cannot confidently confirm the medical diagnosis of acute concussion based on clinical history and physical examination findings alone, consideration should be given to referral to the team neuropsychologist for neuropsychological testing. This testing should be especially considered in patients who report transient and non-specific symptoms following head and neck trauma, such as fatigue or fogginess or those who experience the onset of migraine headaches in the setting of contact sport activities. In circumstances when neuropsychological testing does not reveal results consistent with acute concussion, the definitive diagnosis of acute concussion should remain one of collaborative clinical judgment and is best made by a team of multi-disciplinary TBI experts. Student-athletes with a diagnosed acute concussion should be provided individualized Return-to-School guidance and accommodations as needed.

### Graded Aerobic Exercise Testing

In order to develop an individually tailored rehabilitation program that mitigates the effects of aerobic deconditioning in athletes with acute concussion it is essential to conduct a standardized assessment of exercise tolerance. Over the past decade, the Buffalo Concussion Treadmill Test (BCTT) has emerged as a safe, reliable, valid, and clinically useful tool to measure exercise tolerance in children and adults with acute concussion and PPCS (8, 39, 85–90). Using a modified Balke protocol, concussion patients are exposed to incremental increases in exercise intensity during frequent monitoring of concussion symptoms, heart rate and rating of perceived exertion (RPE). In athletes that are more comfortable exercising on a stationary bike, the authors have developed the Buffalo Concussion Bike Test [for descriptions of both tests see (13)]. In general, acute concussion patients and those with autonomic/physiological post-concussion syndrome will achieve an early symptom-limited threshold on graded aerobic exercise testing. In some cases, however, patients with isolated vestibulo-ocular or cervical spine dysfunction may experience mild vestibulo-ocular or cervicogenic symptoms that typically occur in the later stages of testing when walking at higher speeds that result in greater head translation (10). Importantly, clinical research has demonstrated that the BCTT performed 1–9 days post-injury is safe and is not associated with an increase in concussion symptoms the day following testing (39). In addition, acute concussion patients who demonstrate exercise intolerance at lower heart rate thresholds during the BCTT take longer to recover than those who are capable of exercising to higher heart rate thresholds.

In our clinical practice, the timing of graded aerobic exercise testing depends on a number of factors including the severity of the patient's symptoms, the time since injury, the need for additional supplemental testing, and physician judgment. In collegiate and professional athletes who undergo initial evaluation < 24 h post-injury and who present with a high burden of symptoms, we usually recommend a period of sub-symptom threshold physical and cognitive rest for 24–48 h prior to performing graded aerobic exercise testing. However, in those who present with a lower burden of symptoms < 48 h post-injury and those that present >48 h post-injury we perform graded aerobic exercise testing during the initial medical assessment.

## Individualized Management and Rehabilitation of Acute Concussion

The goals of an individually tailored rehabilitation program are to initiate evidence-based rehabilitation strategies that target the clinico-pathophysiological features responsible for acute concussion symptoms and help maintain the athlete's physical fitness level. The three key pathophysiological processes that are most frequently targeted following acute concussion are: (1) autonomic dysfunction and exercise intolerance; (2) vestibulo-ocular dysfunction; and (3) cervical spine dysfunction (Table 1). Although athletes may manifest clinical features consistent with migraine headaches or post-injury mood or psychiatric disorders, these are much more commonly encountered in patients with PPCS and can often be prevented by optimizing patient management during the acute phase of injury.

**Table 1:** Summary of predominant symptoms, physical examination findings, graded aerobic exercise testing results, treatment recommendations, and multi-disciplinary care for the primary pathophysiological processes underlying acute concussion.

	**Autonomic dysfunction and exercise intolerance**	**Vestibulo-ocular dysfunction**	**Cervical spine dysfunction**
Predominant symptoms	•Mild to moderate global, pounding headache •Dizziness, nausea, fatigue, light and sound sensitivity, difficulty with memory and concentration •Symptoms elicited or exacerbated by physical (and sometimes) cognitive activity	•Mild to moderate headache, eye strain that is elicited or exacerbated by prolonged periods of reading, focusing, or time in complex visuospatial environments •Intermittent blurred vision, diplopia, dizziness, fogginess, motion sensitivity, difficulty focusing or concentrating •Intermittent vertigo during certain head positions (BPPV)	•Mild to moderate, dull, occipital headache that is elicited or exacerbated by activities that require prolonged neck stabilization or movement •Neck pain, stiffness, decreased range of motion, dizziness, fogginess, postural imbalance
Physical examination findings	•Normal physical examination •Elevated resting heart rate •Orthostatic changes in pulse and/or blood pressure accompanied by symptoms	•Objective impairments of convergence, accommodation, smooth pursuits, saccades, and vestibulo-ocular reflex •Impaired balance and postural stability testing •Positive Dix-Hallpike or Supine Roll Test (BPPV)	•Decreased cervical lordosis and range of motion •Sub-occipital and paraspinal neck tenderness •Impaired cervical spine proprioception •Positive cervical dizziness testing
Graded exercise testing results	•Early symptom-limited threshold (i.e., < 70% of age-predicted maximum HR on the BCTT)	•Patients with isolated vestibulo-ocular dysfunction typically do not experience an early symptom-limited threshold	•Patients with isolated cervical spine dysfunction typically do not experience an early symptom-limited threshold.
Targeted treatment	•Sub-symptom threshold aerobic exercise prescription (targeting 80–90% of heart rate achieved during testing)	•Conservative management for 3–5 days •Targeted vestibular and vision therapy •Targeted particle repositioning (BPPV)	•Cervical spine manual therapy and proprioception re-training •Gaze and postural stabilization exercises
Important considerations	•Patients that do not achieve complete recovery with sub-symptom threshold exercise prescription should be screened for migraine headaches and post-injury psychiatric outcomes	•Must clinically exclude structural cervical spine injury before VOR and BPPV testing •Must rule out co-existing neurological and neuro-ophthalmological conditions prior to initiating targeted therapies	•In patients with concerning signs and symptoms, must rule out cervical spine structural injury or mechanical instability prior to initiating targeted therapies
Consulting multi-disciplinary specialists	•Exercise physiologist •Physiotherapist •Athletic therapist	•Vestibular physiotherapist •Neuro-ophthalmologist •Neuro-optometrist	•Cervical spine physiotherapist •Athletic therapist •Chiropractor

*VOR, vestibulo-ocular reflex; BPPV, benign paroxysmal positional vertigo. Updated from (10)*.

### Autonomic Dysfunction and Exercise Intolerance

Accumulating basic science and clinical research suggests that individually tailored sub-symptom threshold aerobic exercise prescription is an effective treatment option for patients with concussion. Animal studies of concussion and TBI suggest that there is a therapeutic window for implementation of individually tailored voluntary aerobic exercise programs that may lead to enhanced clinical recovery through the up-regulation of plasticity-related neuropeptides (91–93) and neurogenesis (94). To date, several observational clinical studies have demonstrated a high rate of symptom improvement and medical clearance to return to sports and other activities among concussion patients with BCTT-confirmed exercise intolerance who are treated with individually tailored sub-symptom threshold aerobic exercise programs (85, 87, 90, 95). In addition, one randomized controlled trial in adolescent mild TBI patients with PPCS (4–16 weeks post-injury) and exercise intolerance demonstrated enhanced symptom improvement over a 6-weeks period in patients prescribed sub-symptom threshold aerobic exercise when compared with those treated with a full-body stretching program (96). Preliminary research suggests that treatment of post-concussion exercise intolerance with sub-symptom threshold aerobic exercise prescription may lead to enhanced clinical recovery through restoration of autonomic nervous system and cerebrovascular functioning (13, 97–99). In addition to these studies in patients with PPCS, there is also preliminary evidence that early sub-symptom threshold aerobic exercise prescription is safe in athletes with acute concussion and may enhance recovery. One randomized controlled trial examined the safety and clinical benefit of structured aerobic exercise in collegiate athletes with acute concussion. They found no differences in length of clinical recovery among patients who engaged in a daily 20-min mild to moderate stationary bike exercise program vs. those managed with standard clinical care (15 days for the treatment group vs. 13 days for the control group) (100). More recently, Leddy and colleagues compared clinical outcomes among adolescent male athletes who were managed conservatively with relative rest vs. those treated with an individually-tailored sub-threshold exercise program (101). Patients treated with exercise experienced a shorter length of recovery (8 days vs. 24 days) and were less likely to develop symptoms persisting >4 weeks (0 vs. 13%).

Given this foundation of emerging evidence, it is our current clinical practice to prescribe all collegiate and professional athletes with acute concussion a supervised and individually-tailored sub-symptom threshold aerobic exercise program consisting of daily 20-min aerobic stationary bike or treadmill work-outs at a heart rate target of 80–90% of the maximum heart rate achieved during graded aerobic exercise testing performed day 1–3 post-injury. Patients with clinical evidence of co-existing vestibulo-ocular and cervical spine dysfunction receive concurrent targeted therapy for these processes as discussed below. Athletes are normally seen every 1–2 days in follow-up when repeat graded aerobic exercise testing is considered to help re-evaluate exercise tolerance and advance the patient's sub-symptom threshold aerobic exercise program. The decision to transition the athlete from the sub-symptom threshold aerobic exercise program back into sport-specific activities including non-contact practices must be made on an individualized basis. For instance, elite ice hockey players can achieve heart rates of 160–180 beats per minute during practice and game play (102, 103). Therefore, we typically ensure that these athletes are capable of achieving a heart rate of 160–180 beats per min during graded aerobic exercise testing without symptom limitation prior to medically clearing them to return to high-intensity strength training and non-contact practice.

### Vestibulo-Ocular Dysfunction

The overall goal of vestibular and vision rehabilitation is to promote the active recovery of post-traumatic visual, vestibular, and somatosensory impairments to restore gaze stabilization, head and eye coordination, balance and sensorimotor processing. To accomplish these objectives, an individualized rehabilitation program must be developed that targets the objective physical examination findings and pathophysiological processes underlying persistent concussion symptoms. While there is strong empirical evidence to support treatment of certain aspects of vestibulo-ocular dysfunction following head trauma, evidence for treatment of other aspects is lacking. For instance, post-traumatic BPPV accounts for 8.5–18% of all BPPV cases and is caused by the dislodgement of otolithic particles into one of the semi-circular canals resulting in recurrent episodes of vertigo that are precipitated by certain head movements and positions (104). There is strong empirical evidence that patients with BPPV should undergo treatment with targeted particle re-positioning that results in a high rate of symptom relief but may require repeat treatment in some cases (49, 105, 106). Similarly, there is moderate to strong empirical evidence that vestibular rehabilitation is safe and effective for patients with peripheral vestibular hypofunction (107, 108). In contrast, the optimal management of impairments in gaze stabilization, sensorimotor integration, and gait and balance deficits that result from injury to central and peripheral components of the vestibulo-ocular system remains unclear (109). Among available randomized controlled trials, Schneider et al. (110) examined the role of cervico-vestibular physiotherapy in sport-related concussion patients presenting with dizziness, neck pain, and/or headache symptoms >10 days post-injury and physical examination findings suggestive of vestibular and cervical spine dysfunction. Treatment with vestibular rehabilitation consisting of habituation exercises, sensorimotor/balance retraining, and otolith repositioning maneuvers as well as cervical spine physiotherapy resulted in an increased proportion of patients obtaining medical clearance to return to sport within 8 weeks compared to those who were managed with usual care (73 and 7%, respectively). More recently, Reneker et al. (111) conducted a randomized controlled trial feasibility study evaluating the effect of individually tailored cervical and vestibular physiotherapy initiated 10 days post-injury in concussion patients with migraine cluster symptoms including dizziness and physical examination findings suggestive of vestibulo-ocular or cervical spine dysfunction. Treatment with individualized and progressive rehabilitation plans was associated with a shorter duration of symptoms (13.5 vs. 17 days) and fewer days until medical clearance (15.5 vs. 26 days) compared to treatment with a non-progressive treatment program.

In addition to targeted vestibular physiotherapy, neuro-optometric vision therapy has also emerged as a potential treatment option for patients with persistent impairments in convergence, accommodation, smooth pursuits, and saccades. Although there is some research to suggest a potential benefit in TBI patients (112, 113) and children with congenital convergence insufficiency (114) no randomized controlled trials evaluating neuro-optometric vision therapy in patients with acute concussion or PPCS have been performed. In one retrospective study of pediatric concussion patients evaluated at a sub-specialty concussion clinic, 24% were found to have an abnormal near point of convergence on physical examination (115). Of these patients, 46% recovered over a median of 4.5 weeks post-injury with conservative management while 41% who underwent vestibular therapy consultation and treatment with a Brock string and pencil push-ups recovered at a median of 11 weeks post-injury. Only 13% required referral to a developmental neuro-optometrist for in-office vision therapy and recovered a median of 23 weeks post-injury.

In our experience, ~30% of collegiate and professional athletes with acute concussion present with subjective and objective clinical evidence of vestibulo-ocular dysfunction. The vast majority of patients achieve normalization of objective physical examination findings within 3–5 days post-injury without any targeted vestibular or vision therapy. Patients who present with persistent vestibulo-ocular symptoms that limit progression through their return-to-sport strategy as well as objective abnormalities on physical examination are referred to a competency-based vestibular physiotherapist for comprehensive assessment and vestibular rehabilitation. Patients who present with post-traumatic BPPV are also immediately referred to a vestibular physiotherapist for assessment and targeted particle repositioning. Lastly, the rare patient who presents with a suspected cranial neuropathy or persistent visual symptoms that do not respond to vestibular rehabilitation is referred to a neuro-ophthalmologist or neuro-optometrist for further assessment and consideration of vision therapy.

### Cervical Spine Dysfunction

Rehabilitation of cervical spine dysfunction following sport-related head and neck trauma should be aimed at reducing cervicogenic headaches and local muscle tenderness, restoring range of motion and optimizing integrated sensorimotor processing within the cervical spine, vestibular, and visual systems. To achieve these objectives, an individualized rehabilitation program must be developed that often includes manual therapy as well as exercises that address objective impairments in passive and active range of motion, neck proprioception, and gaze stabilization. As discussed above, empirical evidence in support of a therapeutic benefit of cervical spine physiotherapy in concussion patients is provided by two randomized controlled trials that demonstrated enhanced clinical recovery in patients with persistent cervical spine and vestibulo-ocular dysfunction treated with targeted cervico-vestibular physiotherapy (110, 111).

In our experience, ~30–60% of collegiate and professional athletes with acute concussion present with subjective and objective evidence of cervical spine dysfunction, which is more common among athletes with a history of previous whiplash-type injuries and those with chronic neck stiffness who receive regular cervical spine manual therapy and chiropractic care. In comparison to vestibular and vision therapy that can lead to significant worsening of vestibulo-ocular symptoms when initiated in highly symptomatic patients within the first few days of injury, it is our experience that the majority of collegiate and professional athletes with acute concussion and associated cervical spine dysfunction report significant improvement in their cervicogenic symptoms with daily, low-intensity manual therapy to the cervical spine and range of motion exercises. Therefore, following medical exclusion of structural and mechanical injury to the cervical spine, we typically refer athletes with acute concussion and clinical evidence of cervical spine dysfunction to an experienced physiotherapist, athletic therapist, or chiropractor to initiate a cervical spine rehabilitation program starting 2–3 days post-injury. In patients with clinical evidence of an associated soft tissue temporo-mandibular joint injury, manual therapy, and exercise programs may also be considered; however, a recent systematic review suggests the quality of the evidence to support these interventions in patients with temporo-mandibular disorders remains low (116).

### Migraine Headaches and Mood Disorders

As outlined in our previous reviews (9, 10), some concussion patients will experience persistent symptoms that are characteristic of migraine headaches and post-injury mood or psychiatric disorders. While these clinical manifestations are much more common among patients who develop PPCS, there are rare cases where these conditions must he addressed following acute concussion in collegiate and professional athletes.

Migraine headaches are diagnosed in patients who experience recurrent attacks of moderate or severe, unilateral, pulsatile, or throbbing headaches that last 4–72 h and are associated with nausea and/or photophobia and may or may not be accompanied by an aura (117). Migraine is the most common primary headache disorder and has a strong genetic basis (118). While migraine is thought to affect 15–20% of the general population, more recent work suggests it may be more prevalent and under-recognized among collegiate athletes (119). Athletes with a history of migraine can experience the acute onset of their stereotypical migraine headaches during athletic competitions or immediately following head trauma (119–121). Furthermore, some athletes with a history of migraine headaches may experience an increase in the frequency or severity of their migraine headaches following acute concussion. Although the pathophysiology of migraine with aura is characterized by cortical spreading depression, alterations in cerebral blood flow, and activation of trigeminal nocioceptive pathways (118, 122–124), the pathophysiology of migraine without aura remains less understood. Consistent with more recent research (125), it is our experience that the majority of collegiate and professional athletes with a history of migraine and acute concussion will make a complete recovery along expected timelines. However, for those experiencing more frequent or severe migraine headaches following acute concussion, we recommend early referral to a headache neurologist to consider a short course of pharmacological treatment that should be discontinued prior to medical clearance (126).

Collegiate and professional athletes with acute concussion can also experience symptoms that reflect disturbances in mood, sleep, and cognitive functioning. Accumulating literature suggests these athletes can experience symptoms of common mental health disorders, such as ADHD, depression, anxiety, and eating and addictive disorders (127–129) in the absence of head injury. Risk factors for the development of post-concussion mood and psychiatric disorders following sport-related concussion may include female sex, a personal, or family history of mood disorders, a greater burden of emotional and overall concussion symptoms at initial assessment and the development of persistent symptoms (130). The development of post-injury mood disturbances following concussion can also be influenced by a number of non-injury factors including sleep, diet, and supplement use, marital, or family dysfunction, and stress as well as other factors that are unique to collegiate and professional athletes, such as stigmatization, performance expectations, and concerns regarding contracts, career security, privacy, and impending sport retirement (127, 131, 132). Athletes with acute concussion should be carefully followed for the development of mood disorders and substance abuse. Given the complexity of underlying factors that contribute to mood disturbances following TBI and the potential for long-term disability and even fatal outcomes, early referral to a sport neuropsychologist, or psychiatrist should be considered to help optimize management of this unique patient population.

### Multi-Disciplinary Medical Clearance

Decisions to medically clear collegiate and professional athletes to return to full contact practice and game play must be made on an individual basis. There is no clear consensus regarding the clinical criteria used to consider patients neurologically recovered and safe to return to full sport activities (133). Preliminary longitudinal studies using advanced techniques suggest that the pathophysiological disturbances that underlie acute concussion may persist beyond symptomatic recovery and may confer a potential risk of vulnerability to repeat trauma (134). Keeping these considerations in mind, it is our current practice to consider collegiate and professional athletes ready to return to full contact practices and games when they are symptom-free or are back to their pre-injury neurological state at rest and during vigorous anaerobic and aerobic exercise, are tolerating non-contact practice without any concussion-like symptoms, have a normal physical examination and have returned to their pre-injury physical fitness level. Student-athletes must have also returned to full-time school without academic accommodations. Finally, it is important that neurocognitive recovery be documented by the team neuropsychologist as outlined by the athlete's standardized concussion protocol (73–75).

In select cases, physicians and multi-disciplinary teams managing collegiate and professional athletes may consider recommending sport retirement due to concerns regarding the short and long-term risks of returning to contact and collision sports. Retirement from sport decision-making in the setting of multiple concussions, multiple concussions in one season, prolonged recovery, PPCS, seizures or epilepsy, neuroimaging evidence of structural brain or spine abnormalities, central cord neuropraxia/SCIWORA, cranial neuropathies, persistent impairments on formal neuropsychological testing, and post-injury mood disorders should be made on an individualized basis (45, 135–139). Given there are limited evidence-based guidelines to inform clinical recommendations in these patients, it is important that physicians and multi-disciplinary experts provide all athletes with the latest information regarding the known and potential risks of repetitive brain injury and sport participation (140) and empower the athlete and other stakeholders (spouses, children, agents, team medical staff, and team management) to come to a collaborative decision that places the highest priority on the athlete's short and long-term health.

### Considerations for Non-elite Athletes

This review was intended to provide guidance for physicians, athletic training staff, and multi-disciplinary experts who provide care to collegiate and professional athletes. In most cases, the care of these athletes is led by experienced physicians with clinical training in concussion and traumatic brain and spine injuries (sports medicine and rehabilitation medicine physicians, neurosurgeons, and sport neurologists). These physicians typically serve as team physicians or consultants, have access to other multi-disciplinary experts in TBI, including neuropsychologists, exercise scientists, physiotherapists, neuro-ophthalmologists, and psychiatrists and work directly with the athlete's team medical and athletic therapy staff. In order to consider active rehabilitation of the pathophysiological manifestations of acute concussion, it is critically important that a definitive medical diagnosis of concussion is confirmed by a physician and that further diagnostic testing (i.e., graded aerobic treadmill testing, neuropsychological testing) and rehabilitation strategies be carried out by the healthcare professionals that are optimally trained and licensed to provide these individual services. The authors are well-aware that over the past decade, there has been an explosion in the number of “concussion clinics” and “certified providers” who are advertising concussion-related services, many of which are doing so without direct on-site access to experienced physicians and healthcare professionals that have competency-based training in TBI related sub-disciplines (141, 142). It must be acknowledged that while collegiate and professional athletes have access to salaried medical professionals who are available to provide daily care for these teams, much of the cost associated with concussion care and rehabilitation received by the general population in the United States and Canada is either charged to third party insurance or borne directly by athletes or their parents. These important differences and inequalities have created an unfortunate entrepreneurial landscape of concussion care whereby individual healthcare professionals with sub-optimal training and resources are marketing elite level or comprehensive concussion care to youth and adult non-elites at considerable private cost. It should be emphasized that ~70–85% of youth and adult athletes will make a complete neurological recovery and successfully return to school and sport activities with conservative management including proper education and guidance (6, 143). When applying this physiological approach to the general concussion/MTBI population, we recommend that care of these patients be carried out under the direct supervision of a physician with experience in concussion and that targeted rehabilitation strategies be implemented in a judicious, cost-conscious, and evidence-based manner by licensed healthcare professionals with competency-based training in neurorehabilitation.

## Conclusions

Sport-related concussion is a common injury sustained among athletes that produces clinical manifestations that result from several unique and overlapping pathophysiological processes. This novel approach to the assessment and rehabilitation of acute concussion builds on our evolving physiological approach to patients with PPCS to offer a comprehensive, evidence-based framework that guides the initial medical assessment and multi-disciplinary management of collegiate and professional athletes with acute concussion. Combining the results of a comprehensive clinical history, physical examination, and graded aerobic exercise testing, this approach allows clinicians to work together to develop an individually-tailored program that promotes the active rehabilitation of acute concussion while minimizing physiological deconditioning. This approach will undoubtedly require further refinement as novel insights into the pathophysiology and evidence-based management of concussion become available in the future.

## Author Contributions

ME, JL, DC, and BW: conception and design of the work, drafting the work and revising it critically for intellectual content, final approval of the version to be published, and agreement to be accountable for all aspects of the work ensuring that questions related to the accuracy and integrity of any part of the work are appropriately investigated and resolved.

### Conflict of Interest Statement

The authors declare that the research was conducted in the absence of any commercial or financial relationships that could be construed as a potential conflict of interest.
